# Distinct Allelic Diversity of *Plasmodium vivax* Merozoite Surface Protein 3-Alpha (*PvMSP-3α*) Gene in Thailand Using PCR-RFLP

**DOI:** 10.1155/2023/8855171

**Published:** 2023-08-11

**Authors:** Kanyanan Kritsiriwuthinan, Warunee Ngrenngarmlert, Rapatbhorn Patrapuvich, Supaksajee Phuagthong, Kantima Choosang

**Affiliations:** ^1^Faculty of Medical Technology, Rangsit University, Pathumthani 12000, Thailand; ^2^Department of Community Medical Technology, Faculty of Medical Technology, Mahidol University, Nakhon Pathom 73170, Thailand; ^3^Drug Research Unit for Malaria (DRUM), Center of Excellence in Malaria Research, Faculty of Tropical Medicine, Mahidol University, Bangkok 10400, Thailand

## Abstract

Considering the importance of merozoite surface proteins (MSPs) as vaccine candidates, this study was conducted to investigate the polymorphism and genetic diversity of *Plasmodium vivax* merozoite surface protein 3-alpha (*PvMSP-3α*) in Thailand. To analyze genetic diversity, 118 blood samples containing *P. vivax* were collected from four malaria-endemic areas in western and southern Thailand. The DNA was extracted and amplified for the *PvMSP-3α* gene using nested PCR. The PCR products were genotyped by PCR-RFLP with *Hha* I and *Alu* I restriction enzymes. The combination patterns of *Hha* I and *Alu* I RFLP were used to identify allelic variants. Genetic evaluation and phylogenic analysis were performed on 13 sequences, including 10 sequences from our study and 3 sequences from GenBank. The results revealed three major types of *PvMSP-3α*, 91.5% allelic type A (∼1.8 kb), 5.1% allelic type B (∼1.5 kb), and 3.4% allelic type C (∼1.2 kb), were detected based on PCR product size with different frequencies. Among all *PvMSP-3α*, 19 allelic subtypes with *Hha* I RFLP patterns were distinguished and 6 allelic subtypes with *Alu* I RFLP patterns were identified. Of these samples, 73 (61%) and 42 (35.6%) samples were defined as monoallelic subtype infection by *Hha* I and *Alu* I PCR-RFLP, respectively, whereas 77 (65.3%) samples were determined to be mixed-allelic subtype infection by the combination patterns of *Hha* I and *Alu* I RFLP. These results strongly indicate that *PvMSP-3α* gene is highly polymorphic, particularly in blood samples collected from the Thai-Myanmar border area (the western part of Thailand). The combination patterns *of Hha* I and *Alu* I RFLP of the *PvMSP-3α* gene could be considered for use as molecular epidemiologic markers for genotyping *P. vivax* isolates in Thailand.

## 1. Introduction

The most recent World Malaria Report reveals that there were 247 million cases of malaria in 84 countries where the disease is prevalent in 2021, as opposed to 245 million cases in 2020. The estimated number of malaria-related deaths in 2021 was 619,000, compared to 625,000 in 2020 [1]. There are five species of *Plasmodium* known to cause malaria in humans: *P. falciparum*, *P. vivax*, *P. ovale*, *P. malariae*, and *P. knowlesi*. Among these species, *P. vivax* is the most geographically widespread species. It is predominant in the region of the Americas and Southeast Asia, comprising 71.5% and 49.9% of estimated malaria cases, respectively [2].

Malaria remains an important public health issue in Thailand, where over 19% of the total population is (13 million people) presently at risk [3]. *P. vivax* is the most prevalent species of human malaria in Thailand and causes about 93% of malaria infections [4]. The forested areas bordering especially western and southern Thailand are malaria-endemic areas [3, 5].

*Plasmodium vivax* has been reported to be responsible for relapsing infection at the liver stage called the hypnozoite [6, 7] and severe complications, such as cerebral malaria, renal failure, acute respiratory distress, and shock [8, 9]. Moreover, chloroquine drug resistance has been reported in vivax malaria, which is mainly endemic in Southeast Asia including the Thai-Myanmar area [10–12].

Genetic diversity has been associated with several characteristics of *Plasmodium species,* including biology, epidemiology, transmissibility by *Anopheles* mosquitoes, immunological responses, as well as drug resistance [13–15]. In endemic areas with high transmission rates, extensively diverse variants are leading to a public health threat because they have likely developed increased virulence and resistance to drugs for their surviving and fitness. Thus, the intensive understanding of parasite genetic diversity plays an important role for designing malaria control programs and developing effective antimalarial drugs and vaccines.

According to treatment failure and multiple drug resistance in malaria disease, the investigation of genetic diversity of *Plasmodium* spp. in endemic areas is significantly considered for antimalarial drugs used in each specific endemic area, particularly in western and southern Thailand. Therefore, the differences among allelic subtypes in western and southern Thailand have been focused in this study.

The *P. vivax* merozoite surface protein 3-alpha (*PvMSP-3α*) is one of the most common polymorphic genes that have been used as molecular markers in studies of genetic diversity, population dynamics [16–19], and potential vaccine candidate [20–22]. *PvMSP-3α* allelic variants have been commonly analyzed by using PCR-restriction fragment length polymorphism (PCR-RFLP) that digested with *Hha* I and *Alu* I restriction enzymes in many different periods and geographical *P. vivax* endemic areas[23–27].

The purpose of this study was to evaluate the genetic polymorphism of *P. vivax* field isolates from malaria-endemic areas of western and southern Thailand. The allelic diversity of *PvMSP-3α* was analyzed by nested PCR-RFLP using two restriction enzymes (*Hha* I and *Alu* I) in order to understand the molecular epidemiology of vivax malaria in Thailand.

## 2. Materials and Methods

### 2.1. Sample Collection

This study was approved by the Ethics Committee of Rangsit University, Thailand (RSUERB2018-026). Blood samples were collected during 2013 to 2018 from malaria-infected patients in western Thailand bordering Myanmar (Kanchanaburi, Mae Hong Son, and Tak provinces) and southern Thailand bordering Malaysia (Yala province). The single infection of *P. vivax* blood samples confirmed by nested PCR was recruited in this study for genetic diversity analysis of the *PvMSP-3α* gene. Two hundred microliters of blood samples were spotted onto filter paper (Whatman 3 MM, Whatman International, Maidstone, England), and the dried blood spots were stored in plastic bags at room temperature prior to DNA extraction with ISOLATE II Genomic DNA Kit (Meridian Bioscience, Inc. USA). The DNA samples were stored at −20°C until analysis. The blood samples were examined by Giemsa stained blood smear microscopy and nested PCR [28] for diagnosis of *P. vivax* infection. The confirmed samples for a single infection of *P. vivax* were recruited for the genetic diversity analysis of the *PvMSP-3α* gene.

### 2.2. Nested PCR of *PvMSP*-*3α* Gene

The allelic types of *PvMSP*-*3α* gene were analyzed using the modified protocol of Bruce et al. [16]. Primary PCR was performed in 20 *µ*l using primers P1 (5′-CAGCAGACACCATTTAAGG-3′) and P2 (5′-CCGTTTGTTGATTAGTTGC-3′); the reaction mixture contained 1 × HSTaq PCR buffer master mix, 0.2 *μ*M each primer, 1 U of Taq HS DNA polymerase (Meridian Bioscience, Inc. USA), and 3 *µ*l of extracted DNA. The conditions were as follows: initial denaturation at 95°C for 5 min and 24 cycles of 94°C for 30 s, 58°C for 30 s, and 72°C for 2 min with the final extension at 72°C for 5 min. Nested PCR was done in 20 *µ*l with primers N1 (5′-GACCAGTGTGATACCATTAACC-3′) and N2 (5′-ATACTGGTTCTTCGTCTTCAGG-3′), using 1 *µ*l of the primary PCR product with 30 cycles of the conditions and reaction mixture similar to the primary PCR. Under UV transilluminator, the PCR product was visualized on 1.2% agarose gel, stained with NEOgreen DNA staining reagent (GELLGENTEK Co., Ltd., Korea). The sample that contained a single PCR fragment size indicating a monoallelic type was considered for the allelic subtype analysis using the RFLP pattern. If a sample presented more than one PCR fragment size, indicating mixed allelic types, then it was excluded from the RFLP analysis.

### 2.3. RFLP Analysis of *PvMSP*-*3α* PCR Products

The allelic subtypes of *PvMSP*-*3α* gene were identified by using the PCR-RFLP technique. Five microliters of each amplified product were digested with *Hha* I and *Alu* I restriction enzymes (NEB, Inc, Beverly, MA, USA) in a total volume of 20 *μ*l as followed by the manufacturer protocol. The digested products were presented by 2.5% agarose gel electrophoresis. The restriction patterns of DNA fragments were photographed for the allelic subtype analysis, and each *Hha* I and *Alu* I RFLP patterns were classified as allelic subtype numbers such as A1, A2, and A3. The allelic subtypes were further diagnosed by the combination patterns of *Hha* I and *Alu* I RFLP.

**Figure 1:**
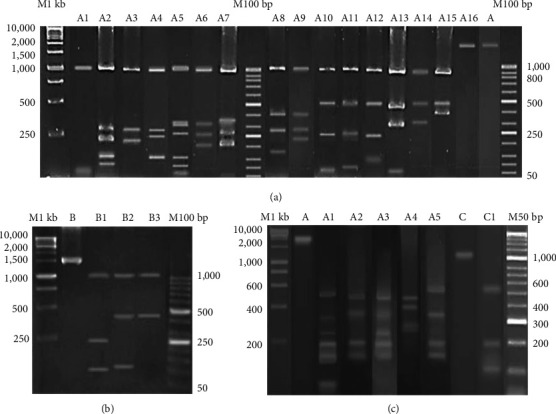
PCR-RFLP analysis of *PvMSP-3α* gene. (a, b) Allelic subtypes identified by *Hha* I RFLP patterns. (c) Allelic subtypes identified by *Alu* I RFLP patterns. A, B, and C indicate the uncut type A, type B, and type C allele variants, respectively. M1kb is 1 kbp DNA marker, M100 bp is a 100 bp DNA marker, and M50 bp is a 50 bp DNA marker.

If the sum of digested DNA fragments was less than or equal to those from uncut PCR, the sample was considered infected with a monoallelic subtype (for example, in Figure 1, the monoallelic subtype showed one or more than single fragment after digestion), named as the monoallelic subtype infection. In case of mixed-allelic subtype infection, the sum of digested DNA fragments was more than the uncut PCR product.

### 2.4. Phylogenetic Analysis of *PvMSP*-*3α* Gene

Ten PCR product samples were analyzed for *PvMSP*-*3α* nucleotide sequence in both directions by the commercial service at 1st BASE, Malaysia. These samples included four collected from Yala province and two each from Kanchanaburi, Mae Hong Son, and Tak provinces. Using basic local alignment search tool (BLAST), phylogenetic relationships among these 10 *PvMSP*-*3α* sequences were compared with the three previously published sequences in the GenBank database, EU430585.1, AF491960.1, and AF093584.2 from Myanmar isolate, Salvador I, and Belem strain, respectively. The phylogenetic tree involving 13 nucleotide sequences was constructed using MEGA7 program with the neighbor-joining method. The bootstrap consensus tree inferred from 1000 replicates.

## 3. Results

A total of 143 single *P. vivax* infected samples were confirmed by both microscopy and PCR. Out of these, 118 samples (82.5%) were successfully amplified for the *PvMSP-3α* gene by nested PCR (Table 1). The distribution of the positive samples in the four provinces of Thailand was 96.3% (78/81) for Yala, 69.2% (18/26) for Kanchanaburi, 63.6% (14/22) for Mae Hong Son, and 57.1% (8/14) for Tak.

### 3.1. Analysis of *PvMSP-3α* Gene by Nested PCR

The genetic diversity of *PvMSP-3α* gene was performed in 118 *P. vivax* isolates. The amplified DNA fragments were differentiated into three allelic sizes: type A (∼1.9 kb), type B (∼1.5 kb), and type C (∼1.2 kb) with corresponding frequencies of 91.5%, 5.1%, and 3.4%, respectively (Table 1). According to the observation of a single band in each sample that reflects a single infection, multiple infections with mixed allelic type were not detected by nested PCR. Two allelic type variants were found in Mae Hong Son (85.7% type A and 14.3% type B), and in Tak (50% type B and 50% type C), whereas in Yala and Kanchanaburi, there were 100% of the allele type A.

### 3.2. Analysis of *PvMSP-3α* Gene by PCR-RFLP

Based on the variation in size and the sum of the digested DNA fragments, the samples could be differentiated into two groups: single and multiple infections. If the sum of the digested DNA fragments was less than or equal to that in the uncut product, the sample was designated as a single infection with a monoallelic subtype. Multiple infections with mixed allelic subtypes were defined when the sum of the digested DNA fragments was more than that in the uncut product.

Among the single infection samples in this study, 16 allelic subtypes A (A1–A16, shown in Figure 1(a)) and 3 allelic subtypes B (B1–B3 shown in Figure 1(b)) of *PvMSP-3α* gene were identified by *Hha* I restriction enzyme, while 5 allelic subtypes A (A1–A5) and 1 allelic subtype C (C1) were classified by *Alu* I restriction enzyme (Figure 1(c)). In Table 2, the subtype A allele with *Hha* I RFLP pattern was found to have the highest frequency (61%; 73/118), and the subtype C allele with *Alu* I RFLP pattern was found to have the lowest frequency (0.8%; 1/118). Besides these, samples with multiple infections that showed multiple bands (named as mixed type) were detected at the frequency of 33.9% (40/118) *Hha* I RFLP pattern, 63.6% (75/118) *Alu* I RFLP pattern, and 65.3% (77/118) combination patterns of *Hha* I and *Alu* I RFLP (Table 2).

**Figure 2:**
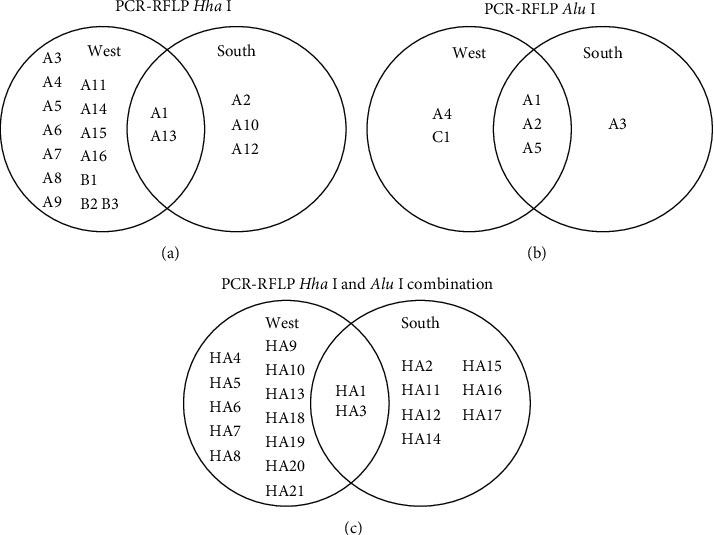
Distribution of *PvMSP-3α* allelic subtypes in western and southern Thailand. (a–c) Allelic variants that bear *Hha* I, *Alu* I, and the combination patterns of *Hha* I and *Alu* I RFLP, respectively.

For western Thailand, allelic subtypes A and B with *Hha* I RFLP patterns were found, in addition to allelic subtypes A and C with *Alu* I RFLP patterns (Figures 2(a) and 2(b)). In southern Thailand, only allelic subtypes A with *Hha* I RFLP pattern and *Alu* I RFLP pattern were found. Among all allelic subtypes analyzed based on combination patterns of *Hha* I and *Alu* I RFLP (HA1-HA21), 2 allelic subtypes (HA1 and HA3) were found in both endemic areas (Figure 2(c)).

### 3.3. Phylogenetic Analysis of *PvMSP-3α* Gene

**Figure 3:**
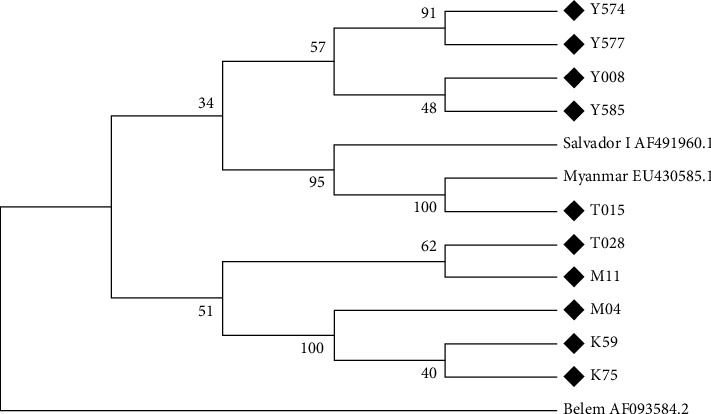
Phylogenetic analysis of *PvMSP-3α* gene. The phylogenetic tree of 13 *PvMSP-3α* sequences was constructed using a neighbor-joining method with the MEGA7 Program. The numbers on the branches indicate the bootstrap proportions (1,000 replicates). Salvador I AF491960.1, Myanmar EU430585.1, and Belem AF093584.2 are the *PvMSP-3α* sequences previously published in GenBank. Y, T, M, and K are the *PvMSP-3α* sequences of *P. vivax* isolates from Yala, Tak, Mae Hong Son, and Kanchanaburi provinces, respectively.

Among the 10 selected isolates, phylogenetic results showed the identity ranging from 90% to 100% with *PvMSP-3α* gene in the GenBank database. The *P. vivax* isolates examined in this study obtained greater than 95% similarity with the isolates from Myanmar, Korea, Thailand, and Mauritania and with GenBank accession numbers EU430583.1, AY266090.1, AY833021.1, and KC935445.1, respectively. The phylogenetic relationships are shown in Figure 3. The selected sequences in this study had a nonspecific relationship to the areas included. From Yala province, isolates were found to be closer to themselves and Salvador I (AF491960.1), while the samples from Kanchanaburi, Mae Hong Son, and Tak provinces were found to have some likeness between themselves and Myanmar (EU430583.1).

## 4. Discussion

Malaria remains a major health risk in Thailand, and *P. vivax* is the most prevalent causative species. Information on the genetic diversity of *P. vivax* is important to understand the dynamics of its populations, support the design of effective malaria control programs, and play an important role in drug and vaccine development. Therefore, this study was carried out to identify *P. vivax* variants and to determine *PvMSP-3α* polymorphism of *P. vivax* isolates collected in malaria-endemic areas of Thailand. *Plasmodium vivax* isolates were collected from western Thailand, bordering Myanmar (Kanchanaburi, Mae Hong Son, and Tak provinces), and southern Thailand, bordering Malaysia (Yala province), during 2013–2018. Nested PCR-RFLP with *Hha* I and *Alu* I restriction enzymes was used to assess the number of distinguishable fragment allelic variants.

Out of 143 samples in the current study, 118 (82.5%) (118/143) were successful for *PvMSP-3α* amplification. The differences in the percentage of successful amplification (57.1% to 96.3%) were probably due to the time and sites of sample collection. Samples from Yala Province had a high successful amplification rate of 96.3% and were mostly obtained during 2016–2018, whereas those from Kanchanaburi, Mae Hong Son, and Tak had lower success rates (57.1%–65.4%) and were mostly collected between 2013 and 2015.

Based on nested PCR, our results show three allelic types of *PvMSP-3α* gene, type A (∼1.9 kb), type B (∼1.5 kb), and type C (∼1.2 kb), in agreement with various worldwide reports. Among these isolates, type A allele had the highest frequency (91.5%), which was consistent with reports from China, India, Pakistan, and Thailand [17, 19, 23–25]. There was no allelic type D and mixed allelic type in this study in contrast to previous reports [26, 29]. Moreover, we found different frequencies of allelic types in each province. Yala and Kanchanaburi revealed only type A, Mae Hong Son contained types A and B, and Tak presented with types B and C. On the contrary, previous reports from Thailand presented all three allelic types in study areas that included western and southern Thailand [23, 24, 30, 31]. These differences may be due to variations in sample collection time and sites of sample collection. In addition, the allelic distribution at the Thai-Malaysia border revealed only type A alleles, but all three allelic types distributed in the Thai-Myanmar border that imply to the origin of type A alleles and genetic diversity in each endemic area.

Our findings with PCR-RFLP demonstrated distinct allelic subtypes of *PvMSP-3α* gene with a high genetic diversity. The results on *PvMSP-3α* polymorphisms from PCR-RFLP with *Hha* I restriction enzyme revealed 19 allelic subtype patterns in 78 *P. vivax* monoallelic subtype samples. The number of allelic subtype variants was more than in previous reports conducted in several different geographical areas including Nepal [32], China [17], Pakistan [29], and Thailand [23, 31, 33].

The lower numbers of allelic subtypes (six allelic subtypes) and faint, indistinct bands of DNA fragments in the gel detected by *Alu* I RFLP patterns accorded with a report by Rice [27] in which in silico digestion with *Alu* I resulted in complicated patterns. Therefore, using the *Hha* I restriction enzyme was suggested for identification of *P. vivax* strain. A high frequency of migration occurs among people along the Thai-Myanmar border, and high polymorphism of *PvMSP-3α* gene was observed in this area [23, 24, 27, 31, 34, 35]. As shown in Figure 2(a), the common subtypes (A1 and A13) in both endemic areas are most likely reflective to the genetic movement of malarial parasites between western and southern Thailand.

The *PvMSP-3α* RFLP with *Hha* I and *Alu* I results showed a high prevalence of mixed subtypes infection (33.9% and 63.6%) among *P. vivax* isolates in Thailand that was higher than in other reports from India, Pakistan, China, and Thailand [17, 18, 25, 26, 29]. These different frequencies might have occurred because of differences in the period of sample collection, geographic area, and detection method. Due to the absence of parasitemia data, this study's limitation lies in the inability to analyze the correlation between parasite density and genotypes of *P. vivax* infection.

In addition, the observation of *P. vivax* variants in different endemic areas most likely indicates different virulence and severity of complications, which has previously been described as an association between multiple genotype infections of *P. vivax* and disease severity [36]. Although several reports have demonstrated the use of PCR-RFLP with various restriction enzymes for detecting malaria multiplicity [16–18, 37, 38], the current study used the combination patterns of *Hha* I and *Alu* I RFLP and detected 21 allelic subtypes, which were more allelic subtypes than classified individually by *Hha* I RFLP or *Alu* I pattern.

## 5. Conclusion

Our study demonstrates a high level of polymorphic of *PvMSP-3α* genes in *P. vivax* isolates that were remarkably distributed in the Thai-Myanmar border area (western Thailand), in the opposite way of Yala (the Thai-Malaysian border area, southern Thailand). The findings of higher numbers of allelic subtypes suggest that the combination patterns of *Hha* I and *Alu* I RFLP could be considered to be used in the molecular epidemiological study of *P. vivax*. An understanding of the allelic diversity among *P. vivax* populations plays an important role in drug and vaccine development for the treatment and control of malaria.

## Figures and Tables

**Table 1:** Allelic frequencies of *PvMSP-3α* gene determined by nested PCR.

Province of Thailand	Total blood sample	*PvMSP-3α* PCR positive sample		Allele type A		Allele type B		Allele type C	
	*N * ^a^	*n*	%	*n*	%	*n*	%	*n*	%
Yala	81	78	96.3	78	100	0	0	0	0
Kanchanaburi	26	18	69.2	18	100	0	0	0	0
Mae Hong Son	22	14	63.6	12	85.7	2	14.3	0	0
Tak	14	8	57.1	0	0	4	50.0	4	50.0
Total	143	118	82.5	108	91.5	6	5.1	4	3.4

^a^The number of confirmed *P. vivax* blood samples.

**Table 2:** Allelic subtype frequencies of *PvMSP-3α* gene determined by PCR-RFLP.

Province	*Hha* I RFLP			*Alu* I RFLP			Combination patterns *of Hha* I and *Alu* I RFLP		
	Allelic subtype	*n*	%	Allelic subtype	*n*	%	Allelic subtype	*n*	%
Yala (southern Thailand) (*n* = 78)	A1	6	5.1	A1	1	0.8	HA1	2	1.7
	A2	5	4.2	A2	11	9.3	HA2	1	0.8
	A10	4	3.4	A3	5	4.2	HA3	2	1.7
	A12	12	10.2	A5	4	3.4	HA11	2	0.8
	A13	23	19.5				HA12	2	1.7
							HA15	3	2.5
							HA16	4	3.4
							HA17	4	3.4
	Mixed type	28	23.7	Mixed type	57	48.3	Mixed type	57	48.3

Kanchanaburi (southern Thailand) (*n* = 18)	A1	4	3.4	A2	7	5.9	HA1	1	0.8
	A4	1	0.8	A5	6	5.1	HA3	3	2.5
	A6	4	3.4				HA5	1	0.8
	A7	1	0.8				HA8	4	3.4
	A11	1	0.8				HA13	1	0.8
	A14	1	0.8				HA19	1	0.8
	A15	2	1.7				HA20	1	0.8
	A16	1	0.8				HA21	1	0.8
	Mixed type	3	2.5	Mixed type	5	4.2	Mixed type	5	4.2

Mae Hong Son (western Thailand) (*n* = 14)	A3	1	0.8	A1	1	0.8	HA4	1	0.8
	A5	2	1.7	A2	4	3.4	HA6	1	0.8
	A8	3	2.5	A4	1	0.8	HA7	1	0.8
	A9	1	0.8	A5	2	1.7	HA9	2	1.7
	A13	1	0.8				HA10	1	0.8
	B3	1	0.8				HA18	1	0.8
	Mixed type	5	4.2	Mixed type	6	5.1	Mixed type	7	5.9

Tak (western Thailand) (*n* = 8)	B1	2	1.7	C1	1	0.8	—	—	—
	B2	1	0.8						
	B3	1	0.8						
	Mixed type	4	3.4	Mixed type	7	5.9	Mixed type	7	6.8

Total (*N* = 118)	A1–A16	73	61.0	A1–A5	42	35.6	HA1-HA22	41	34.7
	B1–B3	5	4.2	C1	1	0.8			
	Mixed type	40	33.9	Mixed type	75	63.6	Mixed type	77	65.3

The mixed genotypes were designated as mixed types.

## Data Availability

All data supporting the findings of this study are included within the paper; however, details of the full data may be obtained from the corresponding author upon request.
